# Acute Liver Failure: Is Acetaminophen the Only Culprit?

**DOI:** 10.7759/cureus.77068

**Published:** 2025-01-07

**Authors:** Scott D McLaughlin, Jason Z Amaral, Joshua Thomas, Keith Z Amaral, Anthony Scalzo

**Affiliations:** 1 Department of Internal Medicine, Advocate Lutheran General Hospital, Park Ridge, USA; 2 Department of Orthopedic Surgery, Texas Children's Hospital, Baylor College of Medicine, Houston, USA; 3 Department of Pediatrics, Sisters of St. Mary (SSM) Health Cardinal Glennon Children's Hospital, Saint Louis University, St. Louis, USA; 4 Department of Pediatrics, Summit Healthcare, Show Low, USA; 5 Department of Pediatrics, Department of Internal Medicine, Division of Toxicology, Sisters of St. Mary (SSM) Health Cardinal Glennon Children's Hospital, Saint Louis University, St. Louis, USA

**Keywords:** acetaminophen toxicity, drug-induced liver injury (dili), ebv-associated hepatitis, ebv liver dysfunction, epstein-barr virus (ebv), fomepizole for acetaminophen toxicity, fomepizole pediatrics, infectious mononucleosis complications, pediatric acute liver failure (alf), pediatric hepatotoxicity

## Abstract

Epstein-Barr virus (EBV) is a common herpesvirus associated with infectious mononucleosis and rare complications such as hepatitis. EBV-associated hepatitis during acute infection may alter liver metabolism, compounding the risk of drug-induced toxicity. We report the case of a 16-year-old female with acute EBV infection who developed severe acetaminophen toxicity after reported use for migraine relief. Her condition was refractory to N-acetylcysteine but improved with the addition of fomepizole, which may have mitigated liver injury by reducing N-acetyl-p-benzoquinone imine (NAPQI) production via CYP2E1 inhibition and suppressing inflammation. This case underscores the complexity of managing pediatric acute liver failure with coexisting hepatic insults, particularly in the presence of viral infections that disrupt liver metabolism, and highlights fomepizole as a potential adjunct in pediatric N-acetylcysteine-refractory acetaminophen toxicity. Further research is warranted to explore the interactions between viral hepatitis and drug-induced liver injury.

## Introduction

Pediatric acute liver failure (ALF) is a rare but life-threatening condition characterized by liver-based coagulopathy, encephalopathy, and hepatocellular injury resulting from the rapid deterioration of hepatocyte function. Acetaminophen toxicity is a common cause of ALF in children [1], and most patients recover with prompt treatment using N-acetylcysteine (NAC) and supportive care. However, children and adolescents with pre-existing liver injury or disease face a heightened risk of severe symptoms due to impaired liver metabolism. Although the incidence of pediatric ALF is not well-defined, it accounts for 10-15% of pediatric liver transplants performed annually in the United States [2].

At therapeutic doses, acetaminophen is primarily metabolized by glucuronidation and sulfation into nontoxic compounds by the liver. At toxic doses, glutathione stores are depleted, saturating the liver’s detoxifying pathways and leading to the oxidation of excess acetaminophen by P450 CYP2E1 into the toxic byproduct N-acetyl-p-benzoquinone imine (NAPQI), the primary mediator of acetaminophen-induced hepatotoxicity [3]. NAC counters acetaminophen toxicity by replenishing glutathione stores, promoting nontoxic metabolism, and detoxifying NAPQI. Recently, fomepizole has gained attention for its potential efficacy in treating acetaminophen toxicity [4-6]. Fomepizole, a competitive inhibitor of alcohol dehydrogenase, also inhibits P450 CYP2E1 in the metabolism phase and c-Jun-N-terminal kinase in the post-metabolic stage, reducing the production of NAPQI and liver inflammation.

Epstein-Barr virus (EBV) is a common herpesvirus infecting up to 90% of the global population [7]. It is particularly prevalent among juveniles and adolescents due to its transmission through saliva, hence the nickname “the kissing disease.” In the United States, seroprevalence increases with age, and one study found that nearly 83% of adolescents aged 18-19 years tested positive for EBV antibodies [8]. During adolescence, approximately 25% of EBV infections progress to infectious mononucleosis, a condition characterized by fever, pharyngitis, and lymphadenopathy, and may present with hepatosplenomegaly and mild elevations in liver enzymes. Less commonly, infectious mononucleosis can progress to hepatitis [9]. While EBV-associated hepatitis is typically self-limited, severe complications such as ALF, though rare, have been reported.

This article was previously presented as a meeting abstract at the 2022 American Medical Association (AMA) Research Challenge on October 20, 2022.

## Case presentation

A previously healthy 16-year-old female with no significant past medical history presented to the emergency department with 1 day of diffuse abdominal pain, nausea, and persistent non-bloody emesis. She reported taking a “handful of acetaminophen” that morning for a migraine. Psychosocial assessment was significant for marijuana usage but otherwise unremarkable for any history of self-harm, suicidal ideation/attempts, anxiety, or depression. She reported a sexual encounter within the last month, used barrier protection, and denied any symptoms consistent with a sexually transmitted infection. The history was otherwise unremarkable.

During the initial examination, she was ill-appearing, mildly tachycardic, and hypertensive with diffuse abdominal pain. There was no guarding, rebound tenderness, appreciable hepatosplenomegaly, or abdominal masses present. Upon reassessment, she became progressively encephalopathic with increasing somnolence and difficulty answering questions.

On admission, Gastroenterology and Toxicology were consulted, and recommended an infectious hepatitis workup with serial monitoring. Laboratory values were significant for K 5.5 mmol/L, HCO3 18 mmol/L, aspartate transaminase (AST) 3,812 IU/L, alanine aminotransferase (ALT) 6,222 IU/L, international normalized ratio (INR) 2.5, prothrombin time (PT) 26.8 sec, and ammonia 119 μmol/L. Comprehensive urine drug screening (UDS) was positive for tetrahydrocannabinol (THC). The patient had undetectable ethanol and salicylate levels. An acetaminophen level was found to be 24 mcg/mL at ~24 h post-ingestion (Table 1). NAC infusion at 12.5 mg/kg/hour was initiated at this time. Infectious and immunological workup included tests for antinuclear antibody (ANA), cytomegalovirus (CMV) IgG/IgM and PCR, EBV PCR, EBV Capsid Ag IgM/IgG, HIV antibody panel, microsomal antibody liver/kidney, smooth muscle antibody, hepatitis A IgM, hepatitis B surface antigen and core IgM, hepatitis C IgG, herpes simplex virus 1/2 (HSV1)/2 PCR, and group A Streptococcus. Of these, EBV PCR was positive, EBV Capsid Ag IgM was elevated (96.4), and EBV Capsid Ag IgG was negative. All other tests were negative.

**Table 1:** Significant laboratory values on patient presentation. K: potassium; HCO_3_: bicarbonate; AST: aspartate transaminase; ALT: alanine aminotransferase; PT: prothrombin time; INR: international normalized ratio

Tests	Results	Normal Ranges
K	5.5 mmol/L	3.5-5.0 mmol/L
HCO_3_	18 mmol/L	22-26 mmol/L
Ammonia	119 μmol/L	9-26 μmol/L
AST	3,812 IU/L	5-40 U/L
ALT	6,222 IU/L	7-56 U/L
PT	26.8 seconds	10.0-14.0 seconds
INR	2.5	0.8-1.2
Ethanol Levels	Undetectable	Undetectable
Salicylate Levels	Undetectable	Undetectable
Acetaminophen Level (24 Hours Post-ingestion)	24 mcg/mL	0 mcg/mL

**Figure 1:**
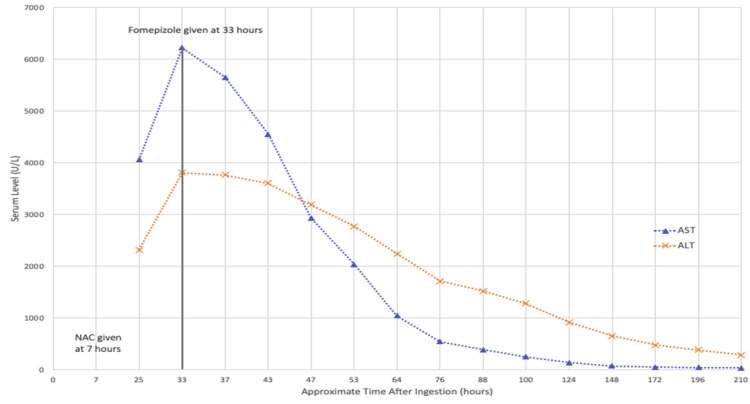
AST and ALT levels throughout the patient’s hospital course. Time at the administration of NAC and fomepizole marked. AST: aspartate transaminase; ALT: alanine aminotransferase; NAC: N-acetylcysteine

Toxicology recommended continuing IV NAC infusion at 12.5 mg/kg/hour as previously initiated with the addition of a fomepizole IV loading dose of 15 mg/kg to prevent additional hepatic injury in the setting of refractory LFT elevations. AST and ALT levels slowly decreased (Figure 1, Table 2) and she was eventually cleared for discharge with instructions for strict outpatient follow-up. The changes in AST and ALT levels over approximate time post-ingestion are presented descriptively.

**Table 2:** Serum AST and ALT values at the approximate time after ingestion of acetaminophen. AST: aspartate transaminase; ALT: alanine aminotransferase

Approximate Time Post-ingestion (hours)	AST (U/L)	ALT (U/L)
25	4,062	2,314
33	6,222	3,812
37	5,657	3,764
43	4,552	3,601
47	2,931	3,192
53	2,033	2,770
64	1,045	2,244
76	543	1,709
88	387	1,519
100	251	1,281
124	139	915
148	74	654
172	47	478
196	41	377
210	37	284

## Discussion

This case highlights the complex interplay between acetaminophen-induced hepatotoxicity and EBV-associated hepatitis in an adolescent patient presenting with ALF. While acetaminophen toxicity is a well-recognized etiology of ALF, the coexisting viral hepatitis likely exacerbated the patient’s condition. Such cases may demand a nuanced approach to treatment to address the compounded insults to liver function.

The patient’s acetaminophen level at approximately 24 hours post-ingestion was consistent with hepatotoxicity. However, the extent of liver enzyme elevation (AST 3,812 IU/L and ALT 6,222 IU/L) and progression to encephalopathy suggest additional contributing factors. EBV-associated hepatitis, confirmed via elevated EBV PCR and positive capsid antigen IgM, likely amplified the toxicity by inducing hepatic inflammation. Viral hepatitis may modulate acetaminophen metabolism by enhancing the activity of CYP2E1, depleting glutathione reserves, and sensitizing hepatocytes to oxidative injury [10-12]. A prospective study of 37 patients hospitalized for viral hepatitis found a positive correlation between plasma acetaminophen levels and the severity of acute hepatitis, suggesting that hepatic inflammation exacerbates acetaminophen toxicity [13]. The researchers concluded that acetaminophen should be avoided in cases of suspected viral hepatitis. This mechanism aligns with findings from preclinical studies demonstrating that inflammatory states render normally safe acetaminophen doses hepatotoxic [11,14]. Clinicians should consider EBV and other viral infections as exacerbating factors in pediatric patients with acetaminophen toxicity, particularly in cases with disproportionate liver enzyme elevations or encephalopathy.

Fomepizole was administered to potentially address the compounded effects of acetaminophen toxicity and hepatic inflammation, given the patient’s refractory LFT elevations and high risk of additional hepatic injury. Its established safety profile, demonstrated in human studies, in-vitro hepatocytes, and animal models, underscores its potential for off-label use in acetaminophen toxicity [4-6,15-17]. By inhibiting CYP2E1, fomepizole reduces the production of NAPQI, while also mitigating downstream inflammatory damage through its action on c-Jun-N-terminal kinase [4,18]. Preclinical studies have demonstrated significant reductions in hepatic necrosis and serum transaminase levels associated with acetaminophen toxicity when fomepizole was used, particularly in combination with NAC [15-19].

Research suggests promising results for fomepizole in treating acetaminophen-induced hepatotoxicity [4-6]. In a prospective case series of 14 high-risk patients treated with fomepizole alongside NAC, no significant liver injury was observed [4]. Notably, five patients (36% of the cohort) had a multiplication product of acetaminophen concentration (in mcg/mL) and aminotransferase levels (in U/L) exceeding 10,000, a threshold predictive of severe hepatotoxicity [4,20]. This case aligns with the findings from these studies, supporting the potential of fomepizole as an effective adjunct in refractory acetaminophen toxicity.

The addition of fomepizole to NAC therapy in this case may have contributed to the patient’s recovery by addressing coexisting hepatic insults. A limitation of this case is the unknown amount of acetaminophen consumed, which limits understanding of the expected rate of elimination and its interaction with EBV-associated hepatitis in contributing to ALF.

Clinicians should maintain a high index of suspicion for viral hepatitis in cases of refractory acetaminophen toxicity in adolescent patients and consider early adjunctive therapies in severe presentations. Further research is needed to evaluate fomepizole’s efficacy, safety, and optimal use in pediatric patients, as well as to explore the interactions between viral hepatitis and drug-induced liver injury.

## Conclusions

The combination of acetaminophen toxicity and EBV-associated hepatitis in this case highlights the complexity of managing pediatric ALF. Fomepizole’s mechanism of action positions it as a potential adjunct to NAC in severe or refractory cases. This report underscores the importance of recognizing coexisting hepatic insults in pediatric acetaminophen toxicity and supports the need for further research into treatment strategies for pediatric ALF.
